# Automatic counting of rapeseed inflorescences using deep learning method and UAV RGB imagery

**DOI:** 10.3389/fpls.2023.1101143

**Published:** 2023-01-31

**Authors:** Jie Li, Yi Li, Jiangwei Qiao, Li Li, Xinfa Wang, Jian Yao, Guisheng Liao

**Affiliations:** ^1^ Hubei Key Laboratory for High-efficiency Utilization of Solar Energy and Operation Control of Energy Storage System, Hubei University of Technology, Wuhan, China; ^2^ Key Laboratory of Biology and Genetic Improvement of Oil Crops, Oil Crops Research Institute of the Chinese Academy of Agricultural Sciences, Wuhan, China; ^3^ School of Remote Sensing and Information Engineering, Wuhan University, Wuhan, China; ^4^ National Lab of Radar Signal Processing, Xidian University, Xi’an, China

**Keywords:** rapeseed, deep learning, unmanned aerial vehicle, attention mechanism, rapeseed inflorescence counting, seed yield

## Abstract

Flowering is a crucial developing stage for rapeseed (*Brassica napus* L.) plants. Flowers develop on the main and branch inflorescences of rapeseed plants and then grow into siliques. The seed yield of rapeseed heavily depends on the total flower numbers per area throughout the whole flowering period. The number of rapeseed inflorescences can reflect the richness of rapeseed flowers and provide useful information for yield prediction. To count rapeseed inflorescences automatically, we transferred the counting problem to a detection task. Then, we developed a low-cost approach for counting rapeseed inflorescences using YOLOv5 with the Convolutional Block Attention Module (CBAM) based on unmanned aerial vehicle (UAV) Red–Green–Blue (RGB) imagery. Moreover, we constructed a Rapeseed Inflorescence Benchmark (RIB) to verify the effectiveness of our model. The RIB dataset captured by DJI Phantom 4 Pro V2.0, including 165 plot images and 60,000 manual labels, is to be released. Experimental results showed that indicators *R^2^* for counting and the mean Average Precision (mAP) for location were over 0.96 and 92%, respectively. Compared with Faster R-CNN, YOLOv4, CenterNet, and TasselNetV2+, the proposed method achieved state-of-the-art counting performance on RIB and had advantages in location accuracy. The counting results revealed a quantitative dynamic change in the number of rapeseed inflorescences in the time dimension. Furthermore, a significant positive correlation between the actual crop yield and the automatically obtained rapeseed inflorescence total number on a field plot level was identified. Thus, a set of UAV- assisted methods for better determination of the flower richness was developed, which can greatly support the breeding of high-yield rapeseed varieties.

## 1 Introduction

Rapeseed (*Brassica napus* L.) is one of the main oil crops, and the development of the rapeseed industry is very important to secure oil supply. The Chinese rapeseed oilseed crop accounts for around 20% of world production. According to the yearbook of the China National Bureau of Statistics, the rapeseed planting area has decreased from 7,316,000 ha in 2010 to 6,765,000 ha in 2020, but the yield per unit area and the total output rose to 2,077 kg/ha and 14.094 million tons in 2020, compared with 1,748 kg/ha and 12.788 million tons in 2010, respectively (National Bureau of Statistics of China, 2021), which shows that the Chinese rapeseed industry has made great achievements in recent years. Yet, rapeseed output is comparatively low considering the consumption for more than 10 consecutive years, as the demand for rapeseed oil increases with the development of the economy and the continuous improvement of consumption level. Affected by the dual effects of rural labor transfer and the shock of imported oil crops, domestic industry development is under enormous pressure (Wang, 2018; Liu et al., 2019). Although China is a major producer of rapeseed oil and rapeseed meal, it mainly relies on inventories and imports to make up for the supply gap (He et al., 2022). Breeding rape varieties with higher oil yield is essential.

Field-based phenotyping plays a vital role in the process of plant breeding for plant performance evaluation (Yang et al., 2017; Jin et al., 2021). The flowering stage of rapeseed lasts as long as 30 days, accounting for almost one-fourth of the growth period. It is a critical period for breeders to analyze the factors that affect the seed yield. The yield components of rape include the number of seeds per pod, the pods, and the weight of each seed (Tayo and Morgan, 1975; Mcgregor, 1981). The number of pods retained at maturity has the greatest effect on the seed yield (Tayo and Morgan, 1975; Mcgregor, 1981; Diepenbrock, 2000; Sonja et al., 2007; Faraji, 2010; Gan et al., 2016; Kirkegaard et al., 2018), which is largely decided by flowering time and flower production that can have potential to turn into pods (Tayo and Morgan, 1975; Faraji et al., 2008; Faraji, 2010; Gong et al., 2018; Kirkegaard et al., 2018; Zhang and Flottmann, 2018; Matar et al., 2020; Zhang et al., 2021). Meanwhile, the amount of rapeseed flowers is closely related to the number of rapeseed inflorescences. As a result, counting rapeseed inflorescences is essential. It is of great significance to explore the correlation between the total number of rapeseed inflorescences of each plot and the seed yield in order to improve the yield of rapeseed. Previous studies highlight the relevance of flowering dates (Han et al., 2021), peak flowering (D’Andrimont et al., 2020), and coverage (Zhang et al., 2021) in seed yield and quality. There is a lack of quantitative description of the number of rapeseed inflorescences. A huge number of rapeseed inflorescences make manual counting impossible. Therefore, an automatic, rapid, and non-destructive method of rapeseed inflorescence counting is useful in plant breeding.

Nowadays, application of satellite remote sensing technology in agriculture has become a trend (Marie et al., 2020). This method obtains multispectral crop information in different periods and spaces from a large area without destroying the crop structure of crops. As a consequence, it has been widely used in precision agriculture (Jin et al., 2017; Khanal et al., 2017; Yang, 2020), yield prediction (Arab et al., 2021; He et al., 2021; Shuai and Basso, 2022), etc. Satellite images have also been successfully applied to rapeseed monitoring. D’Andrimont et al. (2020) combined optical and radar images captured by the Copernicus Sentinel-1 and Sentinel-2 satellite sensors to estimate the flowering timing. Han et al. (2021) utilized the Landsat-8 and Sentinel-1/2 selected from Google Earth to monitor flowering traits. However, low spatial resolution and the period limited the development of satellite images in precision agriculture. Flowering time may be missed because the phenotype of flowers in image changes significantly over a long period of time. Additionally, the size of rapeseed inflorescence is relatively tiny, making it difficult to count tiny objects from a low- resolution satellite image. Recently, the rapid development of unmanned aerial vehicle (UAV) technology provides a new opportunity for continuous acquisition of rapeseed data under different growing stages and it supports different image resolutions. The flexibility and convenience establish an easy way to monitor flowering crops (Kumar et al., 2021; Xu et al., 2021). Wan et al. (2018) employed Red–Green–Blue (RGB) and multispectral images to establish a model to estimate yellow flower number. Zhang et al. (2021) indicated that normalized difference yellowness index-based flowering pixel numbers could estimate flowering intensity by UAV. Sun et al. (2022) extracted spectral traits and structural traits to simultaneously predict wheat yield and grain protein content by multispectral and LiDAR data. Indeed, a lot of plant information is obtained from multispectral data, which is helpful for phenotype analysis. However, multispectral acquisition is greatly affected by weather. The flowering time of different rapeseed materials is inconsistent, and the state of rapeseed flowers changes rapidly in a field. When capturing spectral data, the weather conditions need to be kept as constant as possible and the reliance on it limits multispectral acquisition schemes. It is better to have a UAV scheme that is less affected by the weather.

UAV equipped with RGB cameras (UAV-RGB) has the advantages of higher resolution and less weather affection, which makes it possible to acquire and process large-scale field information conveniently. Recently, combined with the deep learning technology, UAV-RGB system extracted purple leaves (Zhang et al., 2020a), recognized frozen (Li et al., 2022), and estimated stand count (Zhang et al., 2020b) of rapeseed effectively. Many experts are also attracted to study crop counting. Plant counting is regarded as an object- counting task in the computer vision area (Lu and Cao, 2020). For example, Liu et al. (2020a) counted rice to estimate density using the deep learning method. However, these methods discard location information of the plant and the poor explainability limits the counting performances (Lu et al., 2022). More researchers deal with counting as an object detection task, in which target quantity could be estimated from the number of detected bounding boxes. These methods are proven to outperform some traditional machine learning models in counting of maize (Kumar et al., 2021), cotton bloom (Xu et al., 2018), sorghum heads and wheat ears (Lu and Cao, 2020; Lu et al., 2022), etc. Nevertheless, only little attention pays on automatic counting of the rapeseed inflorescences using UAV-RGB because counting rapeseed inflorescences is a challenging task. Inflorescence varies in climate, cultivars, and agricultural management, whose shapes have different degrees of adhesion and occlusion in RGB images. Furthermore, the rape common data set is deficient due to the limitation of rapeseed growth area and time-consuming data annotation, which results in insufficient model training and generates significant challenges to accurate counting.

In order to count rapeseed inflorescences accurately and quickly, we treat the counting task as a detection task. Object detection is a fundamental task in the computer vision area. Current state-of-the-art object detectors are generally divided into two categories, namely, one-stage and two-stage. Faster R-CNN (Ren et al., 2017) is a traditional two-stage network, which proves to be suitable for various plants and plant-organ detection (Madec et al., 2019; Liu et al., 2020b; Xu et al., 2021). These networks have the property of proposal optimization mechanism. Consequently, two-stage operation provides a high accuracy but slow speed and poor real-time performance, which is difficult to meet the requirements of high-throughput and efficient detection. Taking detection as a regression process, the one-stage object detection network does not select candidate regions separately but omits the candidate region generation step. Instead, it integrates feature extraction, target classification, and position regression into one stage for operation. This single- stage network is represented by SSD (Liu et al., 2016), RetinaNet (Lin et al., 2020), and YOLO series (Redmon et al., 2016; Bochkovskiy et al., 2020; Ultralytics, 2021). It has the potential to be faster and simpler (Marie et al., 2020; Yang et al., 2021) but has trailed the accuracy of two-stage detectors (Subramanian and Selvi, 2021). YOLOv5 (Ultralytics, 2021), as an one-stage classic deep recognition end-to-end network model, is the latest version of YOLO series. The model improves the detection speed while maintaining the detection accuracy of existing models. It is one of the optimal choices for the high-throughput detection. Consequently, the improved YOLOv5 model is proposed to enhance the effect of the model in detecting rapeseed inflorescences in the state of dense adhesive occlusion.

This article aims to automatically quantify the total number of rapeseed inflorescences of each plot precisely and quickly. To our knowledge, this is the first time that the deep learning method and UAV-RGB system have been combined to count rapeseed inflorescences. Furthermore, we investigate the correlation between the number of rapeseed inflorescence and the seed yield based on the proposed method. The contributions of this paper are summarized as follows:

• We transformed the rapeseed inflorescences counting as a detection problem and developed an improved YOLOv5 model.
• We built a novel Rapeseed Inflorescence Benchmark (RIB), containing 165 plot images with 60,000 manual labels.
• We assessed the accuracy and robustness of the proposed deep learning algorithms from different rapeseed inflorescences densities, sites, and years.
• We analyzed the correlation between the number of the rapeseed inflorescences and yield and employed the application of inflorescence number in breeding.

## 2 Platform and data preparation

### 2.1 Study area

**Figure 1:**
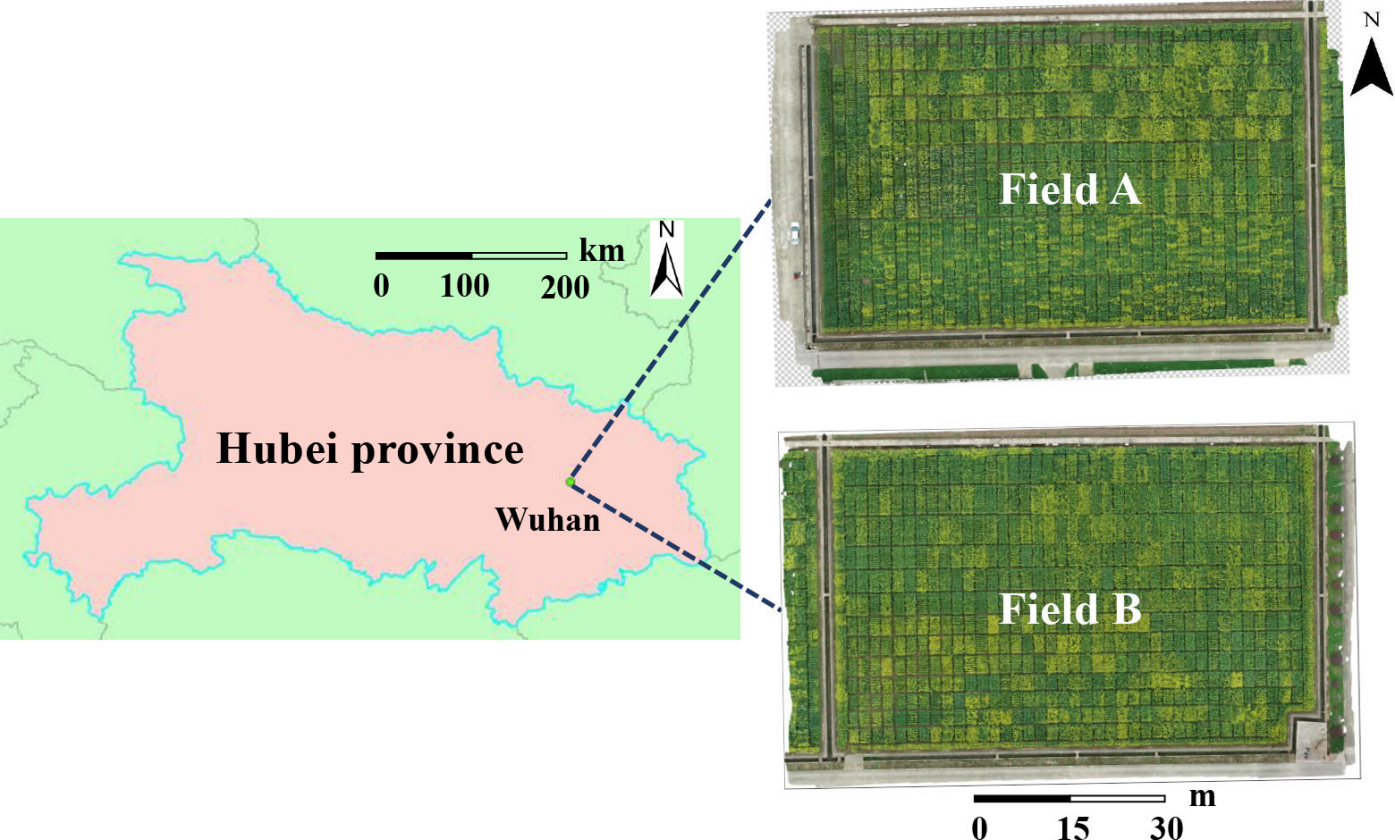
The study area (114.51409E, 30.71047N) at Yangluo Base of Oil Crops Research Institute of the Chinese Academy of Agricultural Sciences, Wuhan, China.

Rapeseed is divided into winter rapeseed (planted at the end of September and harvested in May of the following year) and spring rapeseed (planted at the end of April and harvested in September). Between them, the planting area and output of winter rapeseed account for more than 90% of the country and one-fourth of the world, mainly located in the Yangtze River Basin region. Our study areas belonged to these area. It is located at Yangluo Base (114.51409E, 30.71047N, at an altitude of 24 m, subtropical monsoon climate) of Oil Crops Research Institute of the Chinese Academy of Agricultural Sciences, Wuhan, Hubei Province, China. The details are shown in **Figure 1**. Two experimental fields (rapeseed field A and rapeseed field B) were presented in this study area, of which 252 and 165 plots were chosen, respectively. The training data were composed of the RGB images obtained from rapeseed field B, from which our counting model was obtained. The testing data, consisting of the images acquired from rapeseed field A, were used for testing the robustness of the model with 24 materials planted. In field A and field B, there were two types of plots of different sizes in each field, 8.0 m^2^ (2.0 m long × 4.0 m wide) and 6.0 m^2^ (2 m long × 3 m wide). Experiments were carried out during the flowering stage, from February to May in 2021 and 2022.

### 2.2 Data acquisition

The original image data were collected by DJI Phantom 4 Pro V2.0 ^1^ equipped with an RGB camera. This UAV-RGB system was a consumer-grade drone with a 20- MP (5,472 × 3,648 pixels) image resolution. Rapeseed images were acquired once a week under sunny and partly cloudy conditions in the noon hours (11:00–13:00), local time. Details of the data acquisition environment are shown in **Table 1**. The UAV was set in an automatic acquisition mode to capture images at the speed of 1.9 m/s and a course overlap rate of 75%. Image data acquisition in the study area was completed within 2 h. To obtain images of different scales, we took flight altitudes of 10 and 15 m.

**Table 1:** Image acquisition at Wuhan, Hubei Province, China, in 2021 and 2022.

Year	Acquisition dates	Flight altitude	Field	Temperature	Environment
	February 19	15m	Field A	12°**C**	
	February 26	15m	Fields A and B	14°**C**	
2021	March 3	10m	Fields A and B	21°**C**	11:00 to 13:00, low/middle cloudy or cloudless, wind speed less than 4 m/s, good light conditions
	March 14	10m	Fields A and B	23°**C**	
	March 22	10m	Fields A and B	24°**C**	
	April 5	10m	Field A	15°**C**	
2022	March 1	13m	Field B	20°**C**	

### 2.3 Image dataset

RGB images captured from the UAV were stitched and calibrated automatically by Agisoft PhotoScan ^2^. Thereafter, we obtained six orthophotos of experimental field A and four orthophotos of experimental field B in different periods in 2021. In order to verify the robustness of the model, we acquired a digital orthophoto map of the field in 2022, which would also be applied for further analysis. The deep learning method was a data-driven technique; thus, a large number of samples were needed to train representatives of the detection network model. Rapeseed inflorescence appeared in various forms because of the different flowering time, weather condition, flower size, location, cultivars, postures, colors, occlusions and adhesions, etc. Therefore, enough samples from different flowering stages and conditions were needed to prepare to extract the robust feature.

Details of the construction of the Rapeseed Inflorescence Benchmark (RIB) were as follows. Adobe Photoshop was exploited to crop the field orthophoto into plot sizes. The cropped image resolution was 1,900 × 1,600 for the 8.0- m plot and 1,820 × 680 pixels for the 6.0- m^2^ plot. The RIB dataset included 165 different plot images from field B. The total number of rapeseed inflorescences of each plot varied from 0 to 1,200. Each rapeseed inflorescence was manually labeled with a rectangle using the LabelImg toolbox ^3^. Finally, 60,000 labels were obtained as ground truth for our experiment.

## 3 Materials and methods

**Figure 2:**
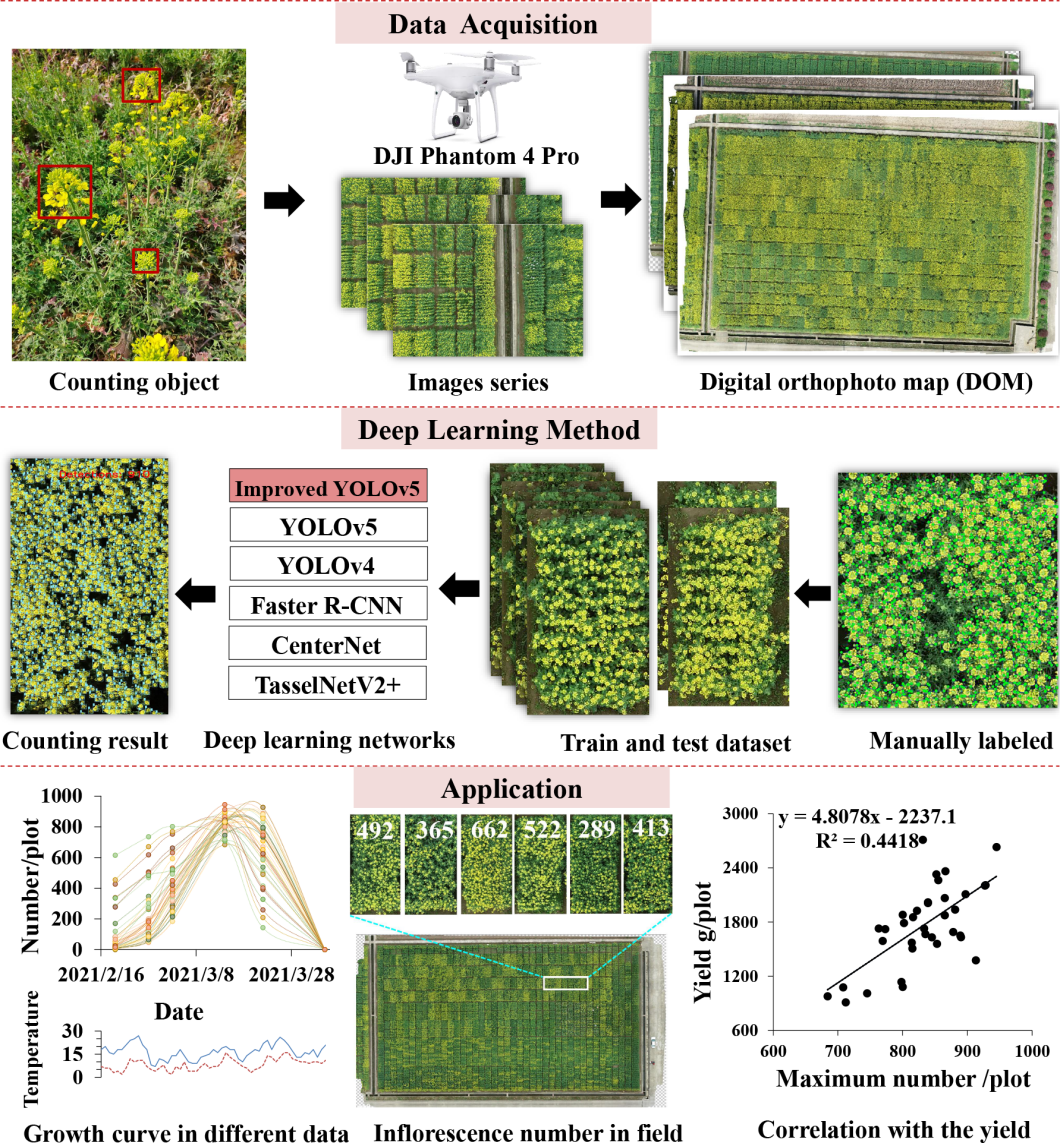
General frame diagram of this study.

In order to address the problem of rapeseed inflorescence counting automatically and efficiently, we proposed a count approach based on deep learning using UAV-RGB imagery. This paper defined a counting object from a top-down perspective. The whole workflow of the proposed approach is presented in **Figure 2**. Firstly, field images were captured during the flowering stage by UAV-RGB imagery. Next, original images were spliced to generate an orthophoto map by Agisoft PhotoScan. Then, the field orthophoto map was cropped to produce plot images by Photoshop ^4^. Afterward, the sample set consisting of all plot images was split to construct train and test datasets to train the network using YOLOv5 combined with the CBAM. After getting the counting results, this paper presented the number difference of rapeseed inflorescences in time series and in field. At last, we investigated the correlation between the total number of rapeseed inflorescences of each plot and the seed yield.

### 3.1 Deep learning method

#### 3.1.1 YOLOv5 network

As a one-stage end-to-end detection network, YOLOv5 transferred detection as a regression process. The network structure consisted of three parts: backbone, neck, and head. First of all, the backbone part was a CSPDarknet53 network for feature extraction and included the structures of Focus, Convolution, Batch Normalization, and SiLU (CBS), C3, and Spatial Pyramid Pooling (SPP). The Focus structure was mainly used for slicing operation. In this way, the width and height information of the pictures was concentrated in the channel space, which could obtain a double downsampling feature map without information loss. The C3 structure is the combination of three convolutional blocks along with the CSP bottleneck. This module is the main module for learning the residual characteristics. The SPP part used different pool kernels to maximize the pooling of the feature map and subsequently spliced feature maps of different scales. This operation combined local features with global features and enriched the expression ability of the feature map. Accordingly, the feature extraction structure that was input with a picture would generate three kinds of scale feature maps. Next, the neck part utilized for feature fusion included Feature Pyramid Network (FPN) and Path Aggregation Network (PAN). FPN fused features through top-down upsampling, whereas PAN transmitted features in a bottom-up pyramid, which enhanced the feature fusion ability of different layers and carried out multiscale prediction. Ultimately, consisting of bounding box loss (regression loss function) and non-maximum suppression (nms), the head structure predicted the category and the location of the object. In addition, YOLOv5 chose Generalized Intersection over Union Loss (GIoU Loss) as the loss function. Three scale feature maps were eventually generated to predict large, medium, and small targets, respectively, in the detection layer of YOLOv5.

YOLOv5 contained four different network structures, namely, YOLOv5s, YOLOv5m, YOLOv5l, and YOLOv5x. The depth and width of the network were controlled by different numbers of residual components in the CSP network, leading to four different structures of YOLOv5. With the increase in depth and width of the network, the ability of learning, feature extraction, and fusion also improved, whereas it paid the cost of longer computing time. Among them, YOLOv5x achieved the highest accuracy but the slowest detection speed. YOLOv5s had the fastest detection speed, three times that of YOLOv5x. As a result, YOLOv5s could better meet the real-time requirements whereas YOLOv5x was able to conform to the condition of high-precision detection.

#### 3.1.2 The improved YOLOv5 network

Input with an image, YOLOv5 would generate bounding boxes for predicting object location in the network. Basically, the rapeseed inflorescence appeared tiny in UAV-RGB images and was in dense overlapping occlusion state. YOLOv5 had the ability to detect small objects. However, it was very difficult to extract enough features due to the variety of the rapeseed inflorescences, especially for adhesion and mutual occlusion. The attention mechanism was imitated from the trait of human vision and widely used in the field of computer vision (Zhu et al., 2019; Hu et al., 2020; Wang et al., 2022a), because it enhanced the key information of the feature and improved the detection accuracy to a certain extent.

The CBAM is a lightweight attention module proposed by Woo et al. (2018), which can perform attention operations in spatial and channel dimensions. It consists of two independent sub-modules, named Channel Attention Module (CAM) and Spatial Attention Module (SAM). CAM learns the weights of different channels and then multiplies them with the weights to enhance the attention to the key channel domain. SAM focuses on the location of the image target. Miao et al. (2022) added the CBAM attention module to YOLOv5 to enhance the feature extraction ability of the backbone network to detect infrared ships. Hoang et al. (2022) utilized the YOLOv5 model with the CBAM attention module to identify various small targets in thermal images. Wang et al. (2022b) integrated the CBAM into the YOLO network and proposed a lightweight one-stage network called the Mobile Ghost Attention YOLO network to improve the performance of the model and the detection of apple leaf diseases. Ye et al. (2022) integrated Convolutional Block Attention Module (CBAM) and Efficient Channel Attention (ECA) into the neck of the latest YOLOv5 network to identify the terminal bud of Chinese fir seedlings in complex backgrounds. The applications mentioned above show that the CBAM attention module is extremely easy to integrate into the YOLOv5 network structure for better representation of objects by focusing on important features and neglecting unnecessary ones without adding too much complexity to the network (Hoang et al., 2022). Thus, to extract the characteristics of the dense rapeseed inflorescences fully, we embedded a CBAM attention mechanism to the YOLOv5 backbone network to attract its attention to local small target features in UAV images to detect dense rapeseed inflorescences well.

**Figure 3:**
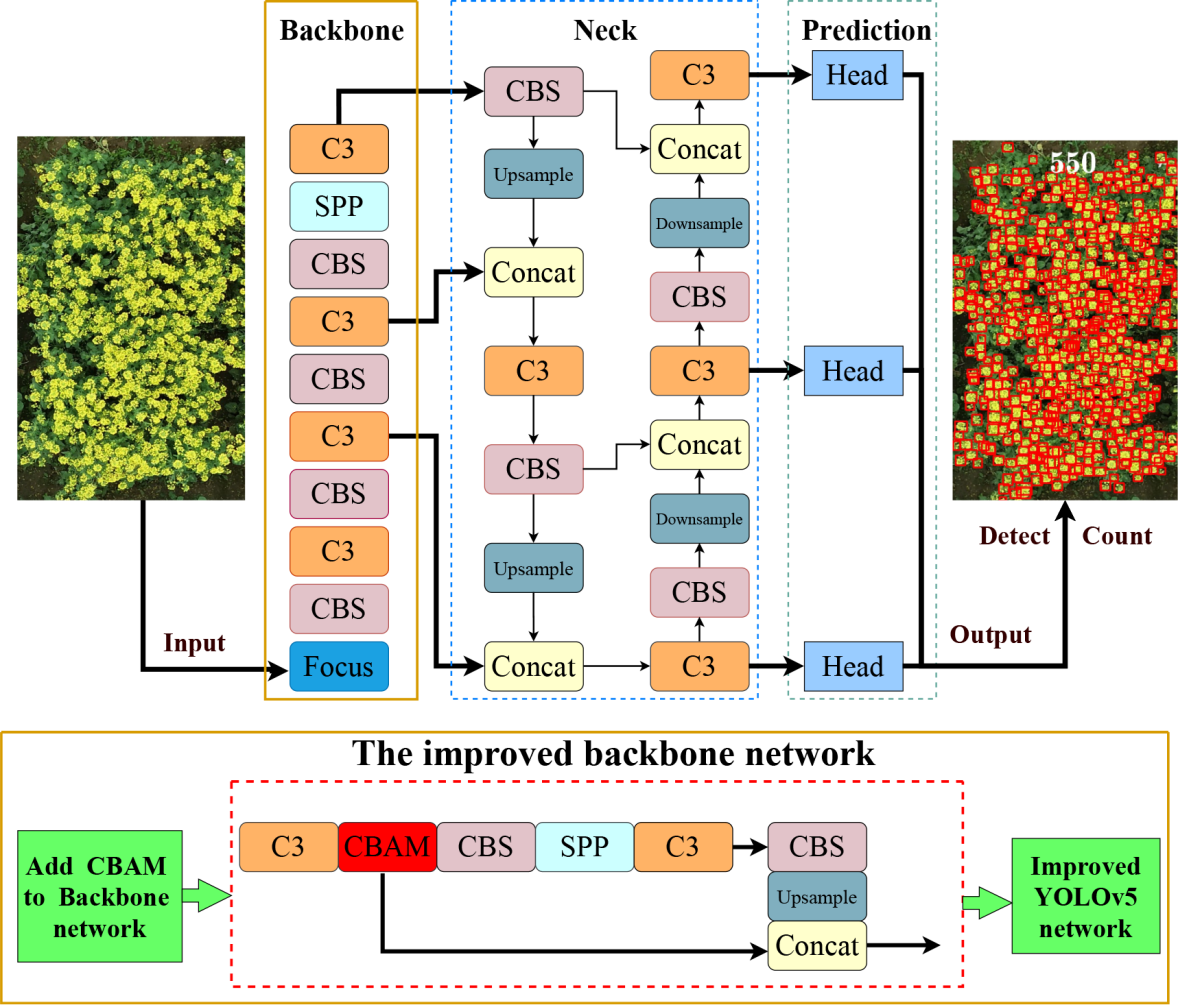
Network structure diagram of the proposed method. The upper part is the YOLOv5 model, and the lower part is the improved backbone network and composition of each module. The improved YOLOv5 generated detection map with the number of rapeseed inflorescences after inputting a rapeseed image.

Different from the above improvement methods, we added the CBAM between C3 and CBS in the backbone. **Figure 3** presents the detailed modification area of YOLOv5-CBAM. The Channel Attention Module of the CBAM enhanced the feature expression of the occluded target, and the Spatial Attention Module highlighted the detecting areas in the feature map. This operation improved the effectiveness and comprehensiveness of feature extraction. It was also convenient for more sufficiency in feature fusion. Moreover, this method worked for the four versions of YOLOv5, making the deployment of YOLOv5-CBAM flexible. The detection results of rapeseed inflorescences were presented in a detection bounding box, whereas the counting results were shown in the counting number.

### 3.2 Evaluation

For the interpretability of counting, this paper selected the detection method for counting. The counting results came from the number of detected bounding boxes. This method could determine the location of rapeseed inflorescence while counting and is helpful in analyzing the reasons for missing or error count. Thus, the accuracy of the counting was closely related to the detection performance.

#### 3.2.1 Detection performance

The paper used Precision, Recall, F1-score, and mean Average Precision (*mAP*) to evaluate the detection performance. Precision is the ratio between the number of accurately predicted samples and the number of actually detected samples in the predicted samples; Recall is the ratio between the number of accurately predicted samples and the total number of samples.

F1-score is a comprehensive evaluation index that assesses the detection accuracy of the model and denotes the harmonic average of Precision and Recall. *mAP* is a measure of the performance of object detectors. The four evaluation metrics are defined as:


(1) $$\text{Precision}=\frac{\text{TP}}{\text{TP}+\text{FP}}$$

(2) $$\text{Recall}=\frac{\text{TP}}{\text{TP}+\text{FN}}$$

(3) $$\text{F}1-\text{score}=2\times \frac{\text{Precision}\times \text{Recall}}{\text{Precision}+\text{Recall}}$$

(4) $$m\text{AP}=\frac{1}{C}\sum_{k=i}^{N}P(k)\Delta R(k)$$

where True Positive (TP) denotes the total number of rapeseed inflorescences correctly predicted in each plot, demonstrating that the correct detection result and the ground truth coincide. False Positive (FP) indicates the total number of rapeseed inflorescences that is incorrectly predicted in each plot, which means that the detection result is rapeseed inflorescence but the ground truth is not. False Negative (FN) is the number of non-flower samples that is incorrectly predicted as rapeseed inflorescences. *C* is the number of categories. Since we only need to detect one category, this value is set to 1. N represents the number of all pictures in the test set. *P*(*k*) means the Precision when *k* pictures can be recognized, and Δ*R*(*k*) denotes the change of the Recall value when the number of recognized pictures changes from *k*-1 to *k*.

#### 3.2.2 Counting performance

To verify the accuracy of the counting, the coefficient of determination (*R^2^*) and the root mean square error (RMSE) are used as evaluation indicators to measure the counting performance of the model. The coefficient of determination means the degree of fit of the regression model and represents the correlation between the predicted value and the true value. The higher the value of *R^2^*, the better the fitting effect of the model is. RMSE denotes the degree of deviation between the true number and the predicted number. The lower the value of RMSE, the better the counting performance of the model is. They are defined as follows:


(5) $$R^{2}=1-\frac{\sum_{i=1}^{n}{\left(m_{i}-c_{i}\right)}^{2}}{\sum_{i=1}^{n}(m_{i}-\;\bar{m}{)}^{2}}$$

(6) $$\text{RMSE}=\sqrt{\frac{1}{n}\sum_{i=1}^{n}{\left(m_{i}-c_{i}\right)}^{2}}$$

where *n* represents the number of plots, *m*_*i*_ and *c*_*i*_ represent the total number of rapeseed inflorescences manually labeled and counted by the model in the *i*th image, respectively, and $\bar{m}$ represents the average number of inflorescences manually labeled in all plots.

## 4 Results and discussion

### 4.1 Network performance

#### 4.1.1 Training details

In this study, the operating system used was the Ubuntu 20.04 operating system with an NVIDIA GTX 3090 GPU. The implementation based on PyTorch 1.8.0 with torchvision 0.9.0. We divided the sample sets into training set, test set, and validation set according to 7:2:1. The initial learning rate and batch size were set to 0.01 and 8, respectively. In order to better optimize the objective function, cosine annealing was utilized to reduce the learning rate in the process of model training. With the increase in the number of iterations, the learning rate first decreased slowly, then accelerated, and then slowly decreased again with the change trend of cosine. Adam optimizer was exploited to train the detection methods. In the training process, the CIoU loss value of the model gradually decreased as the number of iterations increased. The validation loss values changed greatly at the beginning and fell gradually before 150 epochs. After around 400 epochs, both of the loss values tended to be stable and close to a convergent state. Trained for 400 epochs, the final network model of detection was obtained and utilized to detect and count rapeseed inflorescences.

#### 4.1.2 Ablation study

To verify the effect of the CBAM attention mechanism on network detection, we performed ablation experiments in this part. Four network structures of YOLOv5 and the network model with the CBAM attention mechanism were tested on the RIB dataset. From the evaluation results in **Table 2**, we observed that the Precision of the four YOLOv5-CBAM models improved from 0.6% to 1.0% compared with the model without the CBAM. Notably, only the Recall of YOLOv5x-CBAM increased whereas the Recall of the other three YOLOv5 models with the CBAM attention mechanism decreased due to the interaction between Precision and Recall. Compared with the non-CBAM model, the F1-score values of YOLOv5m, YOLOv5l, and YOLOv5x with the CBAM were increased except for the decline of YOLOv5s-CBAM because these two indicators restricted each other in actual situations. Based on the above, *mAP* was exploited to evaluate the detection performance of the model.

**Table 2:** Experimental results of four YOLOv5 network structures before and after improvement.

Model	CBAM	Precision (%)	Recall (%)	F1-score (%)	mAP (%)	*R^2^*	RMSE	Parameters	FPS (f/s)
YOLOv5s	–	90.4	84.8	87.5	90.0	0.943	68.1	**706,3542**	**83.8**
	√	91.4	82.9	86.9	90.9	0.960	56.8	707,4329	83.4
YOLOv5m	–	89.9	85.9	87.9	90.7	0.951	69.2	214,25046	64.8
	√	90.5	85.8	88.1	91.9	0.948	65.1	214,57729	61.7
YOLOv5l	–	91.2	84.7	87.8	90.1	0.962	55.4	472,86710	52.3
	√	**92.2**	83.9	87.9	91.6	0.964	54.3	473,38473	51.2
YOLOv5x	–	89.5	87.0	88.1	91.1	0.954	60.9	882,68374	44.1
	√	90.1	**87.4**	**88.7**	**93.6**	**0.966**	**52.1**	883,43313	44.8

Bold means best value.

After embedding the CBAM, the *mAP* of YOLOv5x, as the largest improvement in the four YOLOv5 structures, was up to 2.5%. For the counting indicators, *R^2^* varied from 0.943 to 0.966. Error and missing detection led to inaccuracy in this value because *R^2^* was an overall statistic. The RMSE decreased more than the model without the CBAM, showing that adding an attention mechanism to the traditional network was effective in improving the counting accuracy of rapeseed inflorescence. The model parameters increased by 10,787, 32,683, 51,763, and 74,940, respectively, but the FPS of the model with the CBAM changed slightly. These means that the target detection time remained basically unchanged after adding a lightweight attention module. From the above analysis, we concluded that it was feasible to count the rapeseed inflorescences using UAV images and deep learning.

**Figure 4:**
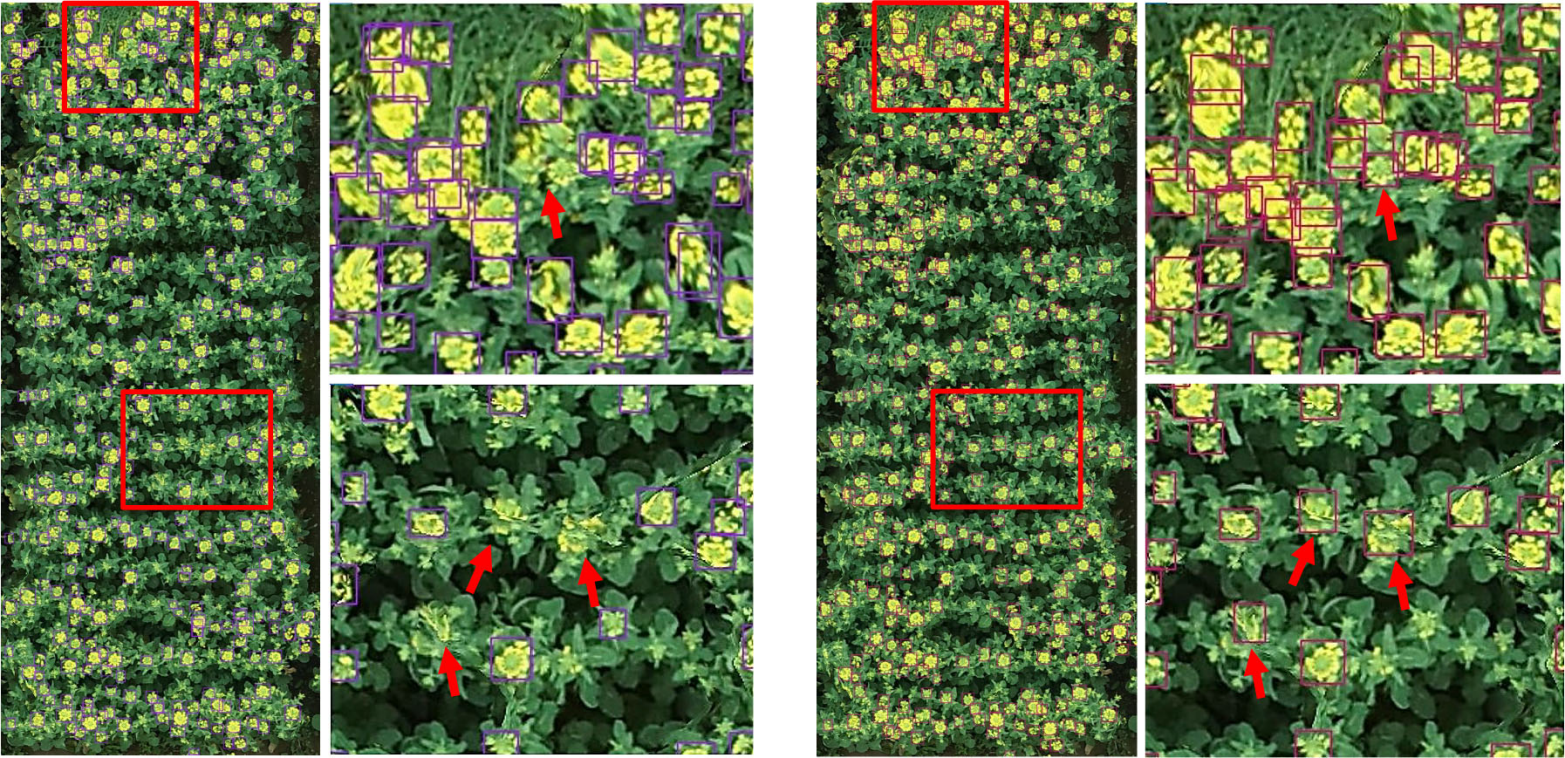
Comparison of detection results between YOLOv5x and YOLOv5x-CBAM. On the left is the YOLOv5x detection result and the partial enlarged view. On the right is the YOLOv5x-CBAM detection result and the partial enlarged view.

For visual comparison, we utilized a similar image to visualize the detecting results in **Figure 4**. The left image and the subimage in **Figure 4** were produced by YOLOv5x, and the right one was generated by YOLOv5x-CBAM. Because the flight height of the UAV was more than 10 m, rapeseed inflorescence presented different degrees of blur in the image. Under these circumstances, even though targets occluded and stuck, both methods could detect correctly most of rapeseed inflorescences. However, YOLOv5x had obvious missed detection in some cases shown in enlarged subimages with red arrows. After adding the attention mechanism, the model could distinguish the difference in adhesion of rapeseed inflorescences and reduce the possibly of missing detection.

### 4.2 Comparison with different networks

To verify the comprehensive performance of the improved network in detection and counting, a study was conducted to compare the performances of several other classical network models, including Faster R-CNN, YOLOv4, CenterNet, and TasselNetV2+. Faster R-CNN (Ren et al., 2017), a traditional two-stage detecting method, utilized the Region Proposal Network (RPN) to generate candidate regions and then combined candidate region generation, feature extraction, target classification, and position regression to achieve an end-to-end structure target detection model. YOLOv4 (Bochkovskiy et al., 2020), a one-stage detecting method and a state-of-the-art object detector, improved and optimized various parts of YOLO series before. Instead of detecting object bounding boxes, TasselNetV2+ (Lu and Cao, 2020) was another plant counting method that directly regressed the number of objects in an image.

**Figure 5:**
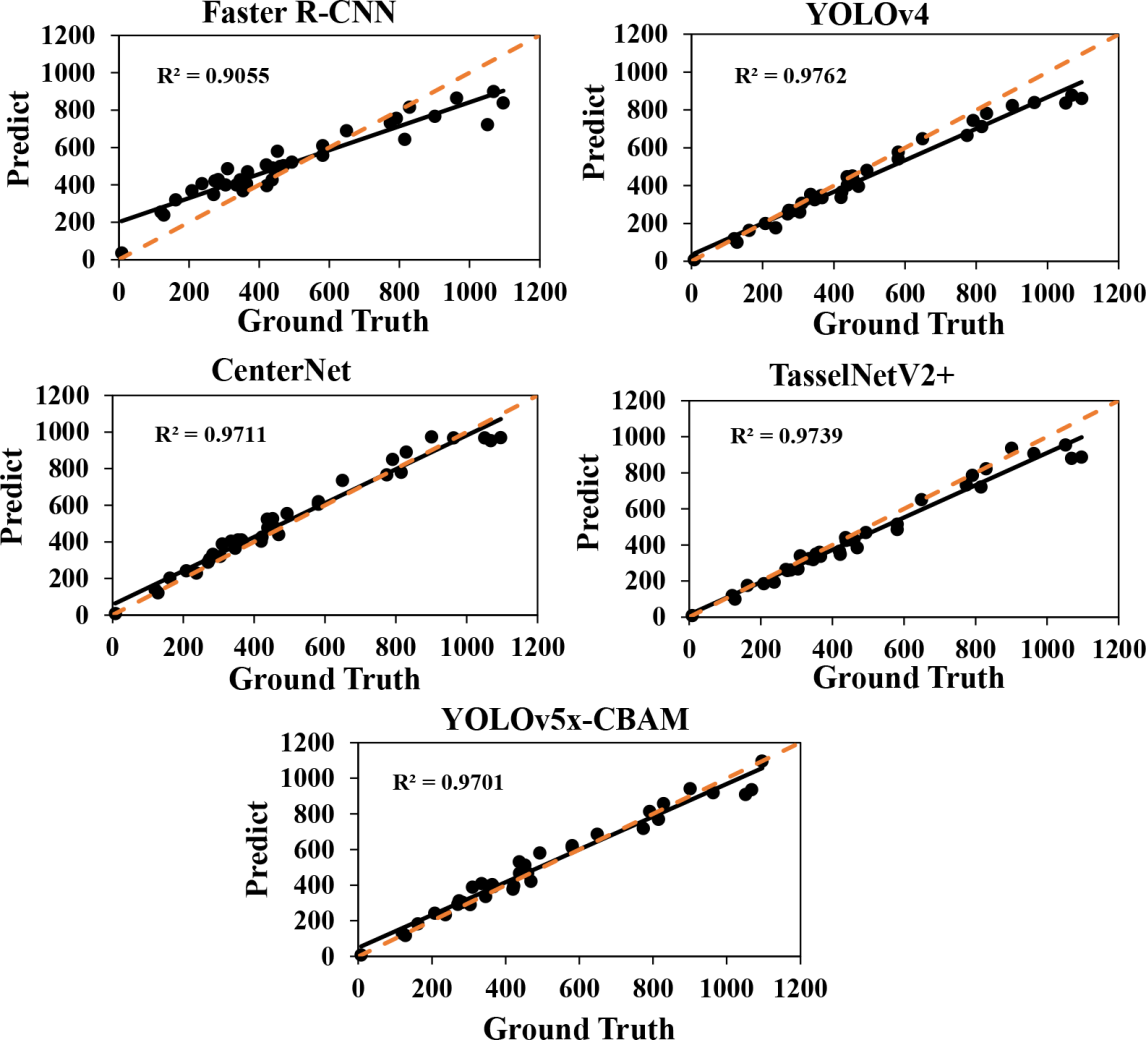
Fitting curves of predicted and true values of five network models. The *R* ^2^ value represents the fitting effect. The orange dotted line represents the 1: 1 fitted line.

As shown in **Table 3**, quantitative results were obtained by using a default parameter setting. The results presented that the proposed approach achieved the best performance in F1-score and *mAP*, both of which were up to 88.7 and 93.6, respectively. Although CenterNet obtained the highest Precision and FPS, its F1-score and Recall were extremely low. It means that the detection speed of CenterNet was the fastest among the five networks, but it failed to correctly predict the rapeseed inflorescence as target in the case of more dense objects and led to a large number of false inspections. The *R^2^* and RMSE value of our method were 0.966 and 52.1, respectively. The counting performance was better than other classical networks. To further prove the validity of the counting, we conducted the experiment to explore the correlation between manual counts and network inferred counts. As shown in **Figure 5**, our method had a strong correlation between manual counts and network model inferred counts. Results in the sixth row of **Table 3** presented that the proposed approach outperformed the state-of-the-art detection methods in RMSE. We could clearly see that our method provided not only interpretable counting results but also detection boxes, providing a basis for subsequent counting improvements.

**Table 3:** Comparison of experimental results between the proposed method and other classical networks.

Methods	Precision (%)	Recall (%)	F1-score (%)	mAP (%)	*R^2^*	RMSE	FPS (f/s)
Faster R-CNN	68.9	53.5	60.0	62.3	0.821	120.2	21.6
YOLOv4	94.0	74.2	83.0	91.4	0.926	180.9	40.5
CenterNet	**95.1**	61.3	75.0	86.3	0.964	53.9	**77.9**
TasselNetV2+	–	–	–	–	0.951	63.3	22.0
The proposed method	90.1	**87.4**	**88.7**	**93.6**	**0.966**	**52.1**	44.8

Bold means best value.

**Figure 6:**
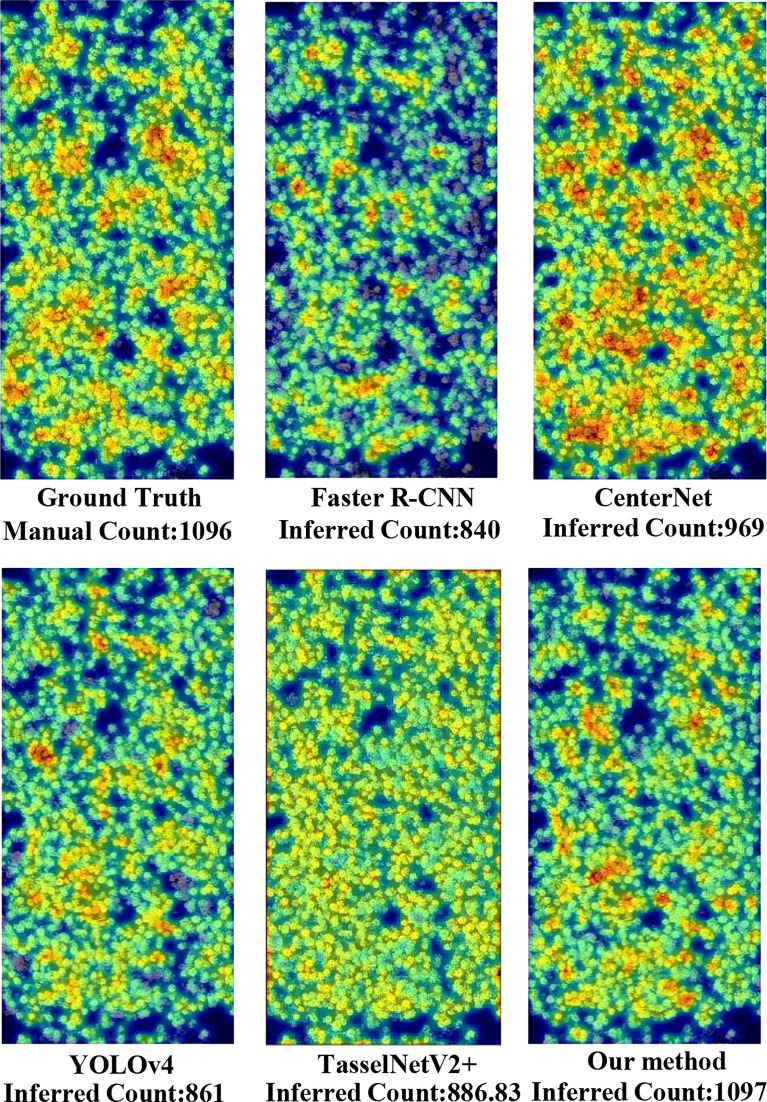
Comparison of visualization results between five networks and the ground truth.

To compare with the ground truth, shown in **Figure 6**, six heatmaps were produced to visualize the counting results. First of all, we output the coordinate value of the prediction box of each traditional network for the same image, then calculated the center point coordinate, and generated the heatmap through Gaussian blur. The heatmap of each traditional network denoted its counting results and the density of rapeseed inflorescences. From the heatmaps, we observed that the detection results of YOLOv4 and the proposed method were closer to ground truth. However, YOLOv4 missed many adhesive rapeseed inflorescence targets, leading to low accuracy of the rapeseed inflorescences counting. For CenterNet, the total counting result was near the ground truth. However, it had duplicate count in the area with dense rapeseed inflorescences and missing count in other areas. This visual result was consistent with the low value of the F1-score indicator shown in **Table 3**. Compared with Faster R-CNN and TasselNetV2+, the detection and counting results of rapeseed inflorescences obtained with our method in the heat map were almost the same as those of the ground truth, indicating that our method was more accurate in detecting and counting rapeseed inflorescences.

### 4.3 The accuracy in different rapeseed densities

**Figure 7:**
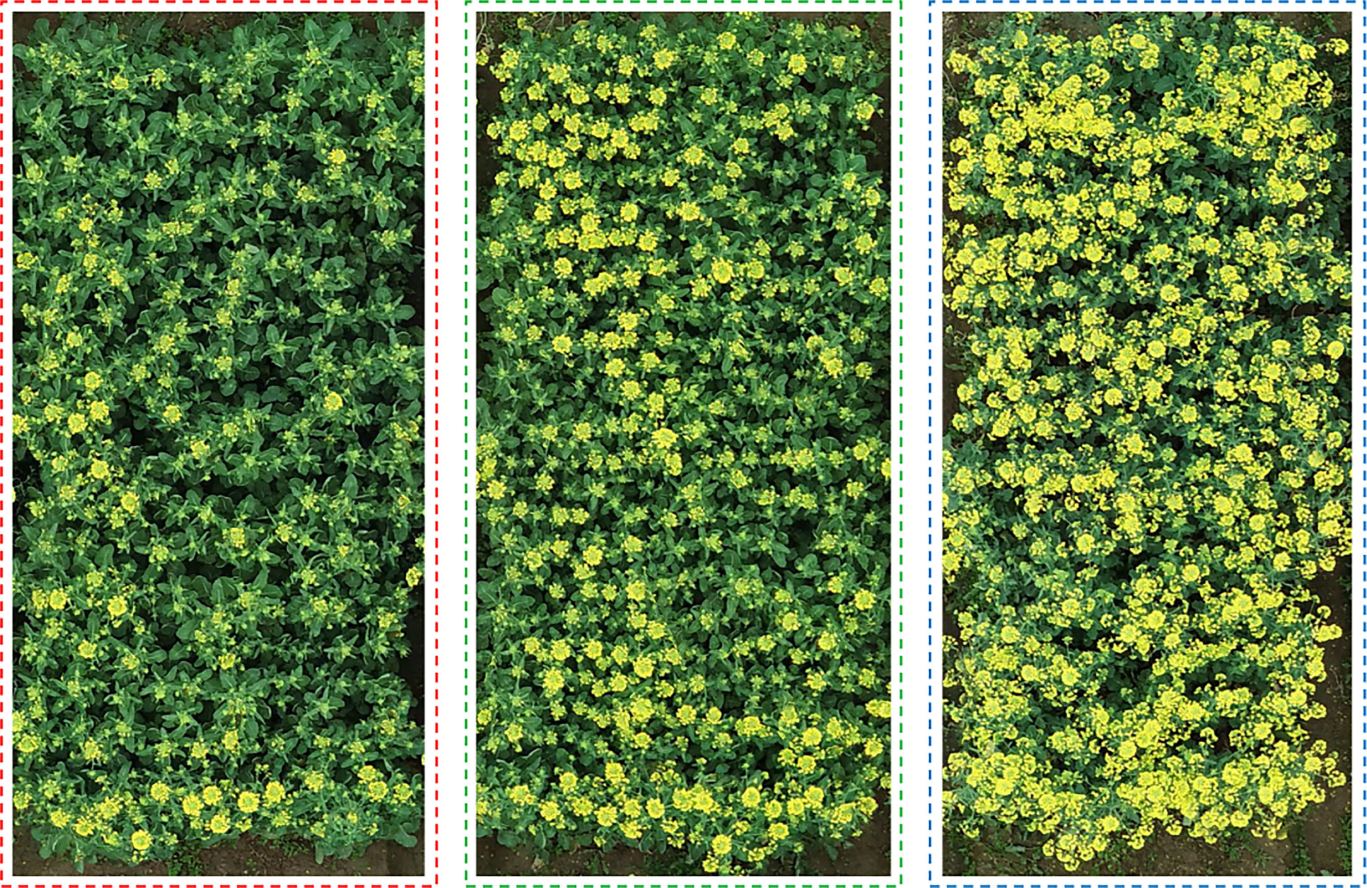
Distribution of rapeseed inflorescence under different densities. The figures on the left, middle, and right represent sparse rape inflorescence, medium-density, and dense rape inflorescences, respectively.

In order to further explore the factors affecting the accuracy of counting, we performed tests using three datasets with different rapeseed densities. As shown in **Figure 7**, we took images of sparse, medium-density, and dense rapeseed from the three datasets that contained 11, 13, and 12 plot images, respectively, as samples. It is observed that green leaves accounted for a large proportion in the sparse rapeseed inflorescence dataset. On the contrary, only yellow rapeseed inflorescences were seen in the dense rapeseed image dataset. The medium-density rapeseed inflorescence dataset contained at least one-third of the visible green leaves from the image and a part of blooming rapeseed.

**Table 4** lists the evaluation results. The *mAP* in the three datasets were 79.6%, 91.5%, and 92.1% and the *R^2^* reached 0.888, 0.930, and 0.927, respectively, meaning that the method proposed by this paper could be applied at any flower stages. However, in the dense rapeseed inflorescence test dataset, the *mAP* decreased to 79.6%. This result further suggested the difficulty of detecting and counting rapeseed inflorescences in occlusion and adhesion areas.

**Table 4:** Experimental results of the proposed method at different densities.

Density	Precision (%)	Recall (%)	F1-score (%)	mAP (%)	*R^2^*	RMSE (%)	Speed (ms)
Dense	85.7	74.0	79.4	79.6	0.888	83.2	12.3
Middle	**92.2**	84.6	88.2	91.5	**0.930**	45.9	12.5
Sparse	89.4	**87.8**	**88.6**	**92.1**	0.927	**30.2**	**11.3**

Bold means best value.

### 4.4 Experiment on the new test site in 2022

**Figure 8:**
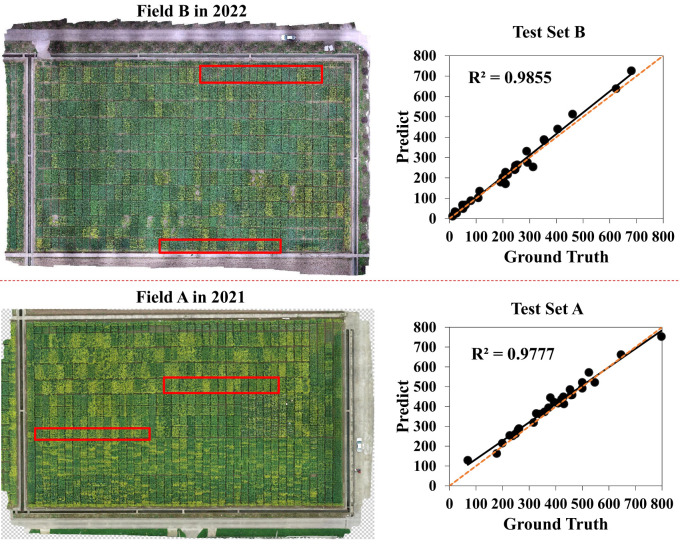
Comparison between inferred counts and manual counts on two novel test sets. Test set B from field B in 2022 is represented by the red box area in the figure’s upper left corner, whereas test set A from field A in 2021 is represented by the red box area in the figure’s lower left corner. The fitting curves of test set B and test set A between the predicted value and the ground truth obtained by the proposed approach in different fields and years are respectively shown on the right. The orange dotted line represents the 1: 1 fitted line.

To validate the robustness and effectiveness of our method, we conducted experiments at the two novel test sets that were called test set A and test set B with manually annotated bounding boxes. Test set A was randomly selected from field A in 2021. Test set B included 30 plot images at the early flowering stage, which we presented in **Figure 8** with the red box area in the digital orthophoto maps. A strong correlation was found between manual counts and predicted counts on these two test sets. As shown in **Figure 8**, the value of *R^2^* on test sets A and B rose up to 0.96 and 0.97, respectively. Our method could well detect and count the rapeseed inflorescences not only in the year of experiment but in the years to come. Therefore, it is applicable to most scenarios of rapeseed inflorescence detection and counting and will meet the needs of conventional farmland management.

### 4.5 The application of counting number

#### 4.5.1 The trend of the number of rapeseed inflorescences in the flowering period

**Figure 9:**
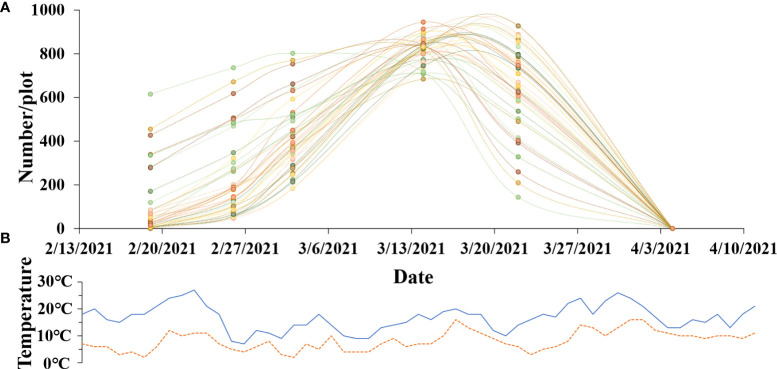
The growth of rapeseed progress during the flowering stage. **(A)** Change curve of the total number of rapeseed inflorescences of each plot. **(B)** Fluctuation curve of maximum temperature and minimum temperature.

The trend of the number change of rapeseed inflorescences is very important for breeding, because we can observe the flowering and withering times of materials. We chose 40 plots from field A randomly and counted the number of rapeseed inflorescences of each plot in six different periods by utilizing the proposed method. The data of six periods are shown in the x-axis, beginning in February 13, 2021, and ending in April 9, 2021. As shown in **Figure 9A**, the y-axis is the rapeseed inflorescence number. The range varied from 0 to 1000. **Figure 9B** presents the minimum and maximum temperatures of the flowering period in the study area. The maximum temperature during the day was around 20C, and the minimum temperature at night was around 5C, which was suitable for the growth of rapeseed. The paper exploited dotted lines of different colors to fit the change in the number of rapeseed inflorescences in different plots in the flowering period. From **Figure 9A**, we observe that most of the plots bloomed around February 18 and withered after March 20, and the peak flowering time was around mid and late March.

#### 4.5.2 The differences between the quantity of the rapeseed inflorescences in field

**Figure 10:**
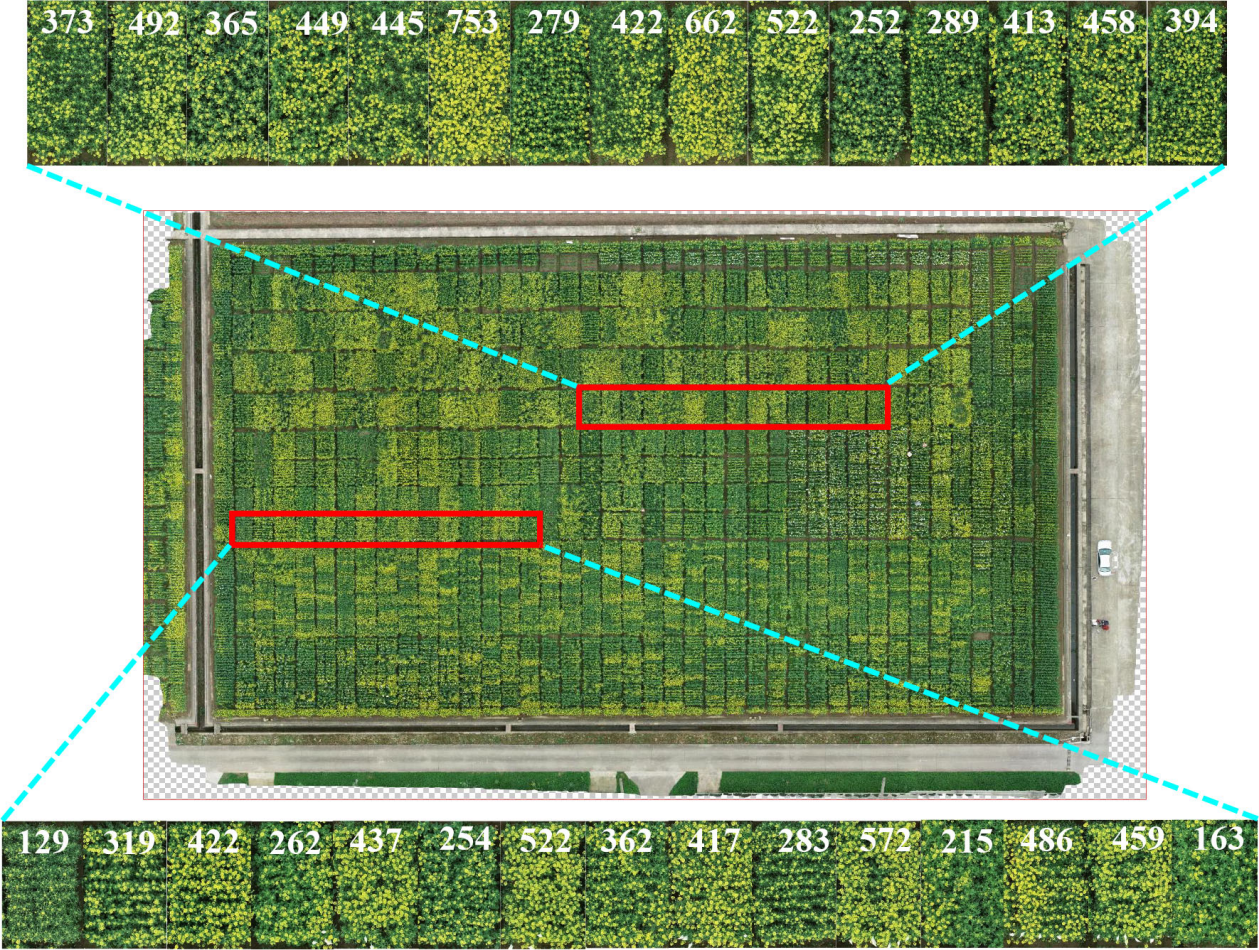
Experimental results of 30 test plots randomly selected in the new field obtained by the proposed method during the flowering stage in field A, March 3, 2021. The red box represents test plots, and the enlarged subimage represents count results.

In addition to observing one material in time order, we compared the differences between the quantity of the rapeseed inflorescences in field. One picture of test field A in March 3, 2021, was chosen. The number of rapeseed inflorescences in different plots was quickly and quantitatively obtained by the proposed method. The counting results are shown in **Figure 10**. The red box and its enlarged subimage included 30 plot images, and each number marked on these images represents its corresponding counting result. Compared with visual observation, the counting results were consistent with the growth trend of the rapeseed. We quantified the change of flowering stage by automatic counting, which brought convenience for breeders to analyze the material performance in the flowering period.

#### 4.5.3 The number of rapeseed inflorescences and the seed yield

The number of rapeseed inflorescences in the flowering period is an important predictor for its seed yield. To further study the effect of rapeseed inflorescence number on yield, we analyzed the correlation between the number of rapeseed inflorescences and the seed yield. We used the data from five flowering periods of field A. The total number of rapeseed inflorescences predicted by the proposed network model for each plot and the corresponding seed yield were recorded to explore the correlation between them. The coefficient of determination (*R* ^2^ ) was exploited to reflect the fitting degree of the linear regression model, representing the interpretation degree of the total number of rapeseed inflorescences of each plot to the seed yield.

**Figure 11:**
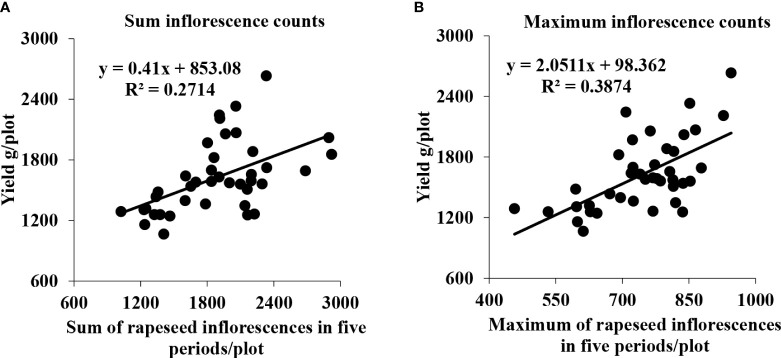
Scatter plots between the seed yield and the total number of rapeseed inflorescences of the representative plots. **(A)** Correlation between the seed yield and sum of all rapeseed inflorescences of each plot in five periods. **(B)** Correlation between the seed yield and maximum count of all rapeseed inflorescences of each plot in five periods.

Initially, we utilized some representative plots that included two different area sizes to perform a regression analysis between the sum and max of the total number of rapeseed inflorescences of each plot in the five periods and the seed yield. As shown in **Figure 11**, the *R* ^2^ value in **Figures 11A, B** reached 0.2714 and 0.3874, respectively. The *R* ^2^ of the sum of the rapeseed inflorescences in the five periods was slightly lower than that of the max of the rapeseed inflorescences in the five periods. It might be that we collected data at a low frequency. There was a sequence in flowers blooming, different materials, and planting methods leading to different flowering times that led to the omission of many rapeseed inflorescences in the whole flowering period.

**Figure 12:**
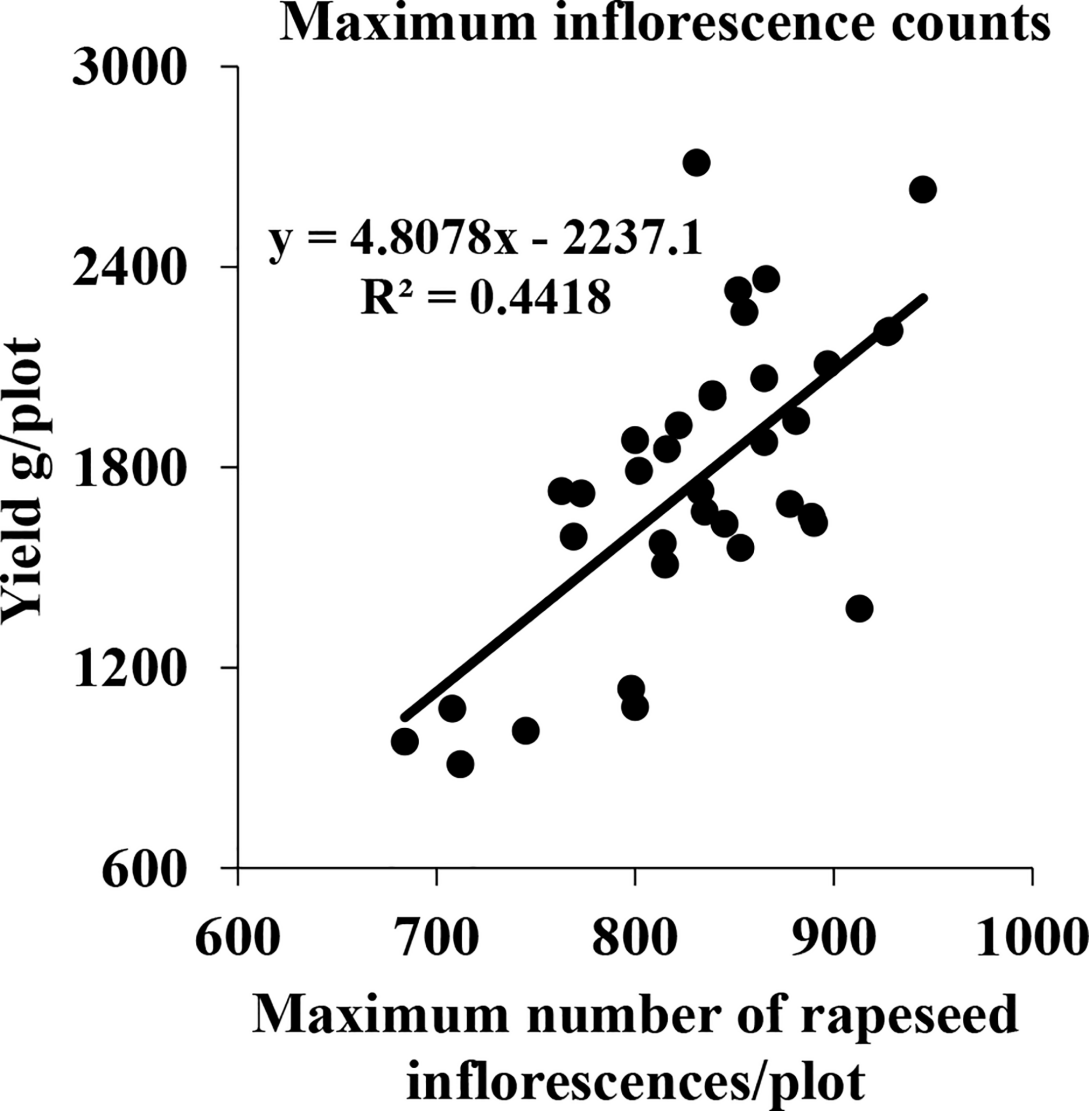
Correlation between the seed yield and maximum number of all rapeseed inflorescences of each plot in five periods in field A in 2021.

Although flower formation at different stages contributed less or more, they might still have the potential to affect the seed yield. Consequently, some plots with equal plant area, good growth, and relatively high yield were selected to further investigate the correlation between the maximum of the total number of rapeseed inflorescences of each plot in the five periods and the seed yield. The results are shown in **Figure 12**. The *R^2^* value, reaching 0.4418, indicated that there was a significant correlation between the maximum of the total number of the rapeseed inflorescences of each plot in the five periods and the seed yield.

## 5 Conclusion

The flowering stage of rapeseed is a critical period for breeders to analyze the factors that affect the seed yield. In this paper, we apply the YOLOv5-CBAM method to quantify the total number of rapeseed inflorescences of each plot automatically, precisely, and quickly. The results show that the detecting precision is up to 91.7% in the RIB dataset. Additionally, we verify the robustness of the proposed method in the datasets of sparse, medium-density, and dense rapeseed inflorescences and found that our method is suitable for rapeseed inflorescences with different densities. Moreover, we conduct comparative experiments on several classical counting networks, including YOLOv4 for one-stage detection networks, Faster R-CNN for two-stage networks, CenterNet for a detection method without an anchor box, and TasselnetV2+ for a counting method without detection boxes. The experimental results verify the effectiveness of the proposed method. In fact, due to its strong robustness and effectiveness, our method suits most scenarios and can also be applied to the detection and counting of other crops that are manually labeled, such as apple, wheat, cotton, and sunflower.

However, inflorescences on the same rapeseed plant have different performances in different periods. In this paper, only several periods of rapeseed are selected as representatives, and a problem of insufficient sample performance appears. Subsequent studies will consider sample diversities and enrich the benchmark. On the other hand, the accuracy of detection directly affects the effect of counting. In the case of dense rapeseed inflorescences, the detecting accuracy is only 85.7%, showing that a lot of room for improvement still remains to be done in this case, in which we will further consider designing more attention modules for the rapeseed inflorescence detection to increase the accuracy.

Finally, we perform regression analysis between the total number of rapeseed inflorescences of each plot and the seed yield to find out their correlation. Under the multiperiod factor, there is a significant correlation between the maximum of the total number of rapeseed inflorescences of each plot in five periods and the seed yield. The total number of rapeseed inflorescences obtained with our method in each plot is the primary factor that affects the yield. However, the seed yield is also affected by many other factors, such as seed weight, seed number per silique, photosynthetic capacity of silique wall, and the abiotic resistance of plants. Thus, we will focus on the pod stage of rapeseed in the future. Lodging after flowering and UAV combined with multi-spectrum are considered to estimate pods. Then, combining the different factors was done to conduct multiple regression analysis to predict yield more accurately.

Based on the nature of the rapeseed growth process, the number of rapeseed inflorescences gradually increases until the flowers withered. During this period, the number of rapeseed inflorescences is closely related to the seed yield. However, we can only capture images of the rapeseed inflorescences in six different periods of flowering. Considering the insufficient data of the whole flowering period, we will increase the sampling frequency in flowering period to better fit the change curve of the number of rapeseed inflorescences in future work.

## Data availability statement

Publicly available datasets were analyzed in this study. This data can be found here: https://github.com/LYLWYH/Rapeseed-Data.

## Author contributions

All authors made significant contributions to this manuscript. JL, YL, and JQ performed field data collection and wrote the manuscript. JQ and LL designed the experiment. JY, XW, and GL provided suggestions on the experiment design. All authors read and approved the final manuscript.

## Footnotes

1
https://www.dji.com/cn/phantom-4-pro-v2

2
https://www.agisoft.com/

3
https://github.com/tzutalin/labelImg

4
https://www.adobe.com/cn/products/photoshop.html

## References

[B1] Arab S. T. Noguchi R. Matsushita S. Ahamed T. (2021). Prediction of grape yields from time-series vegetation indices using satellite remote sensing and a machine-learning approach. Remote Sens. Applications: Soc. Environ. 22, 100485. doi: 10.1016/j.rsase.2021.100485

[B2] Bochkovskiy A. Wang C. Liao H. M. (2020). YOLOv4: Optimal speed and accuracy of object detection. arXiv 2004, 10934. doi: 10.48550/arXiv.2004.10934

[B4] D’Andrimont R. Taymans M. Lemoine G. Ceglar A. Yordanov M. Velde M. V. D. (2020). Detecting flowering phenology in oil seed rape parcels with sentinel-1 and -2 time series. Remote Sens. Environ. 239, 111660. doi: 10.1016/j.rse.2020.111660 32184531 PMC7043338

[B3] Diepenbrock W. (2000). Yield analysis of winter oilseed rape (Brassica napus l.): a review. Field Crops Res. 67, 35–49. doi: 10.1016/S0378-4290(00)00082-4

[B5] Faraji A. (2010). Flower formation and pod/flower ratio in canola (Brassica napus l.) affected by assimilates supply around flowering. Int. J. Plant Production 4, 271–280. doi: 10.22069/IJPP.2012.710

[B6] Faraji A. Latifi N. Soltani A. Rad A. H. S. (2008). Effect of high temperature stress and supplemental irrigation on flower and pod formation in two canola (Brassica napus l.) cultivars at mediterranean climate. Asian J. Plant Sci. 7, 343–351. doi: 10.3923/ajps.2008.343.351

[B7] Gan Y. Harker K. N. Kutcher H. R. Gulden R. H. Irvine B. May W. E. . (2016). Canola seed yield and phenological responses to plant density. Can. J. Plant Sci. 96, 151–159. doi: 10.1139/cjps-2015-0093

[B8] Gong Y. Duan B. Fang S. Zhu R. Wu X. Ma Y. . (2018). Remote estimation of rapeseed yield with unmanned aerial vehicle (UAV) imaging and spectral mixture analysis. Plant Methods 14, 70. doi: 10.1186/s13007-018-0338-z 30151031 PMC6102863

[B9] Han J. Zhang Z. Cao J. (2021). Developing a new method to identify flowering dynamics of rapeseed using landsat 8 and sentinel-1/2. Remote Sens. 13, 105. doi: 10.3390/rs13010105

[B10] He W. Li J. Wang X. Lin Q. Yang X. (2022). Current status of global rapeseed industry and problems, countermeasures of rapeseed industry in china. China Oils Fats 47, 1–7. doi: 10.19902/j.cnki.zgyz.1003-7969.210430

[B11] He X. Yang L. Li A. Zhang L. Shen F. Cai Y. . (2021). Soil organic carbon prediction using phenological parameters and remote sensing variables generated from sentinel-2 images. Catena 205, 105442. doi: 10.1016/j.catena.2021.105442

[B12] Hoang V. P. Ninh H. Hai T. T. (2022). “CBAM-YOLOv5 for infrared image object detection,” in Artificial intelligence and machine learning in defense applications IV (Berlin, Germany: SPIE), 116–127. doi: 10.1117/12.2640690

[B13] Hu J. Shen L. Samuel A. Sun G. Wu E. (2020). Squeeze-and-excitation networks. IEEE Trans. Pattern Anal. Mach. Intell. 42, 2011–2023. doi: 10.1109/TPAMI.2019.2913372 31034408

[B14] Jin X. Liu S. Baret F. Hemerlé M. Comar A. (2017). Estimates of plant density of wheat crops at emergence from very low altitude UAV imagery. Remote Sens. Environ. 198, 105–114. doi: 10.1016/j.rse.2017.06.007

[B15] Jin X. Zarco-Tejada P. J. Schmidhalter U. Reynolds M. P. Hawkesford M. J. Varshney R. K. . (2021). High-throughput estimation of crop traits: A review of ground and aerial phenotyping platforms. IEEE Geosci. Remote Sens. 9, 200–231. doi: 10.1109/MGRS.2020.2998816

[B16] Khanal S. Fulton J. Shearer S. (2017). An overview of current and potential applications of thermal remote sensing in precision agriculture. Comput. Electron. Agric. 139, 22–32. doi: 10.1016/j.compag.2017.05.001

[B17] Kirkegaard J. A. Lilley J. M. Brill R. D. Ware A. H. Walela C. K. (2018). The critical period for yield and quality determination in canola (Brassica napus l.). Field Crops Res. 222, 180–188. doi: 10.1016/j.fcr.2018.03.018

[B18] Kumar A. Desai S. V. Balasubramanian V. N. Rajalakshmi P. Guo W. Balaji Naik B. . (2021). Efficient maize tassel-detection method using UAV based remote sensing. Remote Sens. Applications: Soc. Environ. 23, 100549. doi: 10.1016/j.rsase.2021.100549

[B19] Li L. Qiao J. Yao J. Li J. Li L. (2022). Automatic freezing-tolerant rapeseed material recognition using uav images and deep learning. Plant Methods 18, 1–13. doi: 10.1186/s13007-022-00838-6 35027060 PMC8756653

[B20] Lin T. Goyal P. Girshick R. He K. Dollar P. (2020). Focal loss for dense object detection. IEEE Trans. Pattern Anal. Mach. Intell. 42, 318–327. doi: 10.1109/TPAMI.2018.2858826 30040631

[B21] Liu C. Feng Z. Xiao T. Ma X. Zhou G. Huang F. . (2019). Development, potential and adaptation of chinese rapeseed industry. Chin. J. Oil Crop Sci. 41, 485–489. doi: 10.7505/j.issn.1007-9084.2019.04.001

[B22] Liu L. Lu H. Li Y. Cao Z. (2020a). High-throughput rice density estimation from transplantation to tillering stages using deep networks. Plant Phenomics 2020, 1375957. doi: 10.34133/2020/1375957 33313541 PMC7706318

[B23] Liu W. Anguelov D. Erhan D. Szegedy C. Reed S. Fu C. . (2016). “SSD: Single shot multibox detector,” in European Conference on computer vision (Las Vegas, NV, USA: Springer), 21–37. doi: 10.1007/978-3-319-46448-0\s\do5(2

[B24] Liu Y. Cen C. Che Y. Ke R. Ma Y. (2020b). Detection of maize tassels from uav rgb imagery with faster r-CNN. Remote Sens. 12, 338. doi: 10.3390/rs12020338

[B25] Lu H. Cao Z. (2020). Tasselnetv2+: A fast implementation for high-throughput plant counting from high-resolution RGB imagery. Front. Plant Sci. 11. doi: 10.3389/fpls.2020.541960 PMC7750361 33365037

[B26] Lu H. Liu L. Li Y. Zhao X. Wang X. Cao Z. (2022). Tasselnetv3: Explainable plant counting with guided upsampling and background suppression. IEEE Trans. Geosci. Remote Sens. 60, 1–15. doi: 10.1109/TGRS.2021.3058962

[B27] Madec S. Jin X. Lu H. Solan B. D. Liu S. Duyme F. . (2019). Ear density estimation from high resolution RGB imagery using deep learning technique. Agric. For. Meteorology 264, 225–234. doi: 10.1016/j.agrformet.2018.10.013

[B28] Marie W. Frédéric J. Grgory D. (2020). Remote sensing for agricultural applications: A meta-review. Remote Sens. Environ. 236, 111402. doi: 10.1016/j.rse.2019.111402

[B29] Matar S. Kumar A. Holtgräwe D. Weisshaar B. Melzer S. (2020). The transition to flowering in winter rapeseed during vernalization. Plant Cell Environ. 44, 506–518. doi: 10.1111/pce.13946 33190312

[B30] Mcgregor D. I. (1981). Pattern of flower and pod development in rapeseed. Can. J. Plant Sci. 61, 275–282. doi: 10.4141/cjps81-040

[B31] Miao L. Li N. Zhou M. Zhou H. (2022). “CBAM-Yolov5: improved yolov5 based on attention model for infrared ship detection,” in International conference on computer graphics, artificial intelligence, and data processing (ICCAID 2021) (Guangzhou, China: SPIE), 564–571. doi: 10.1117/12.2631130

[B32] National Bureau of Statistics of China (2021). China Statistical yearbook-2021 (Bei Jing: China Statistics Press).

[B33] Redmon J. Divvala S. Girshick R. Farhadi A. (2016). “You only look once: Unified, real-time object detection,” in Proceedings of the IEEE conference on computer vision and pattern recognition (CVPR) (Amsterdam, The Netherlands: IEEE), 779–788. doi: 10.1109/CVPR.2016.91

[B34] Ren S. He K. Girshick R. Sun J. (2017). Faster r-cnn: Towards real-time object detection with region proposal networks. IEEE Trans. Pattern Anal. Mach. Intell. 39, 1137–1149. doi: 10.1109/TPAMI.2016.2577031 27295650

[B35] Shuai G. Basso B. (2022). Subfield maize yield prediction improves when in-season crop water deficit is included in remote sensing imagery-based models. Remote Sens. Environ. 272, 112938. doi: 10.1016/j.rse.2022.112938

[B36] Sonja I. Cane S. Zoran D. Ana M. J. Mirjana J. Ljupčo J. (2007). Interrelationship between yield and yield related traits of spring canola (Brassica napus l.) genotypes. Genetika 39, 325–332. doi: 10.2298/GENSR0703325I

[B37] Subramanian P. Selvi S. T. (2021). Detection of maturity stages of coconuts in complex background using faster r-CNN model. Biosyst. Eng. 202, 119–132. doi: 10.1016/j.biosystemseng.2020.12.002

[B38] Sun Z. Li Q. Jin S. Song Y. Xu S. Wang X. . (2022). Simultaneous prediction of wheat yield and grain protein content using multitask deep learning from time-series proximal sensing. Plant Phenomics 2022, 9757948. doi: 10.34133/2022/9757948 35441150 PMC8988204

[B39] Tayo T. O. Morgan D. G. (1975). Quantitative analysis of the growth, development and distribution of flowers and pods in oil seed rape (Brassica napus l.). J. Agric. Sci. 85, 103–110. doi: 10.1017/S0021859600053466

[B40] Ultralytics (2021). Available at: https://github.com/ultralytics/yolov5.

[B41] Wan L. Li Y. Cen H. Zhu J. Yin W. Wu W. . (2018). Combining uav-based vegetation indices and image classification to estimate flower number in oilseed rape. Remote Sens. 10, 10127–10134. doi: 10.3390/rs10091484

[B42] Wang G. Zhai Q. Liu H. (2022a). Cross self-attention network for 3d point cloud. Knowledge-Based Syst. 247, 108769. doi: 10.1016/j.knosys.2022.108769

[B43] Wang H. (2018). New-demand oriented oilseed rape industry developing strategy. Chin. J. Oil Crop Sci. 40, 613. doi: 10.7505/j.issn.1007-9084.2018.05.001

[B44] Wang Y. Wang Y. Zhao J. (2022b). MGA-YOLO: A lightweight one-stage network for apple leaf disease detection. Front. Plant Sci. 13. doi: 10.3389/fpls.2022.927424 PMC9441945 36072327

[B45] Woo S. Park J. Lee J.-Y. Kweon I. S. (2018). “CBAM: Convolutional block attention module,” in Proceedings of the European conference on computer vision (ECCV) (Munich, Germany: Springer International Publishing), 3–19. doi: 10.1007/978-3-030-01234-2\s\do5(1

[B46] Xu R. Li C. Paterson A. H. Jiang Y. Sun S. Robertson J. S. (2018). Aerial images and convolutional neural network for cotton bloom detection. Front. Plant Sci. 8. doi: 10.3389/fpls.2017.02235 PMC5820543 29503653

[B47] Xu R. Li C. Paterson A. H. Jiang Y. Sun S. Robertson J. S. (2021). Estimates of maize plant density from uav rgb images using faster-RCNN detection model: impact of the spatial resolution. Plant Phenomics 3, 181–196. doi: 10.34133/2021/9824843 PMC8404552 34549193

[B48] Yang B. Gao Z. Gao Y. Zhu Y. (2021). Rapid detection and counting of wheat ears in the field using YOLOv4 with attention module. Agronomy 11, 10127–10134. doi: 10.3390/agronomy11061202

[B49] Yang C. (2020). Remote sensing and precision agriculture technologies for crop disease detection and management with a practical application example. Engineering 6, 528–532. doi: 10.1016/j.eng.2019.10.015

[B50] Yang G. Liu J. Zhao C. Li Z. Huang Y. Yu H. . (2017). Unmanned aerial vehicle remote sensing for field-based crop phenotyping: Current status and perspectives. Front. Plant Sci. 8. doi: 10.3389/fpls.2017.01111 PMC5492853 28713402

[B51] Ye Z. Guo Q. Wei J. Zhang J. Zhang H. Bian L. . (2022). Recognition of terminal buds of densely-planted chinese fir seedlings using improved yolov5 by integrating attention mechanism. Front. Plant Sci. 13. doi: 10.3389/fpls.2022.991929 PMC9589298 36299793

[B52] Zhang H. Flottmann S. (2018). Source-sink manipulations indicate seed yield in canola is limited by source availability. Eur. J. Agron. 96, 70–76. doi: 10.1016/j.eja.2018.03.005

[B53] Zhang J. Xie T. Yang C. Song H. Jiang Z. Zhou G. . (2020a). Segmenting purple rapeseed leaves in the field from UAV RGB imagery using deep learning as an auxiliary means for nitrogen stress detection. Remote Sens. 12, 1403. doi: 10.3390/rs12091403

[B54] Zhang J. Zhao B. Yang C. Shi Y. Liao Q. Zhou G. . (2020b). Rapeseed stand count estimation at leaf development stages with uav imagery and convolutional neural networks. Front. Plant Sci. 11. doi: 10.3389/fpls.2020.00617 PMC7298076 32587594

[B55] Zhang T. Vail S. Duddu H. S. N. Parkin I. A. P. Guo X. Johnson E. N. . (2021). Phenotyping flowering in canola (Brassica napus l.) and estimating seed yield using an unmanned aerial vehicle-based imagery. Front. Plant Sci. 12. doi: 10.3389/fpls.2021.686332 PMC8249318 34220907

[B56] Zhu X. Cheng D. Zhang Z. Lin S. Dai J. (2019). “An empirical study of spatial attention mechanisms in deep networks,” in Proceedings of the IEEE/CVF international conference on computer vision (ICCV) (Seoul, South Korea: IEEE), 6688–6697. doi: 10.1109/ICCV.2019.00679

